# A confused ECG with multiple rhythms caused by atrial premature contractions: A case report

**DOI:** 10.1097/MD.0000000000006997

**Published:** 2017-12-15

**Authors:** Yang Hongliang, Yu Ming, Zhao Qini, Si Daoyuan, Tong Yaliang, Wang Ying, He Yuquan

**Affiliations:** Department of Cardiology, China-Japan Union Hospital of Jilin University, Changchun, China.

**Keywords:** arrhythmia, electrocardiogram, electrophysiology study, premature atrial contraction

## Abstract

**Introduction:**

Atrial premature contractions (APCs) are commonly encountered in clinical practice. The APCs may influence heart conduction system and induce other arrhythmia. The disorder of atrioventricular conduction is related to electrophysiological phenomena, difficult to understand and diagnose.

**Case report:**

We presented a 15-year-old male patient whose baseline electrocardiogram (ECG) was confused with multiple rhythms. Electrophysiological study results showed sinus rhythm with nonconducted APCs in bigeminal rhythm. Nonconducted APCs were blocked without H wave. Some APCs conducted to ventricle with longer AH interval and HV interval. When APCs were abolished by radiofrequency ablation, this patient was free from any arrhythmia during follow-up.

**Conclusion:**

We considered that the basic rhythm of the baseline ECG was sinus rhythm with atrial bigeminy rhythm and narrow QRS extrasystoles (junctional); some APCs were blocked and some APCs conducted to ventricle with aberrant QRS complexes. The phenomenon of baseline ECG was caused by the APCs.

## 1 Introduction

Atrial premature contractions (APCs) are commonly encountered in clinical practice. The APCs may influence heart conduction system and induce other arrhythmia. The disorder of atrioventricular conduction is related to electrophysiological phenomena, difficult to understand and diagnose. Its understanding may require an electrophysiological study. We presented a case with confused baseline electrocardiogram (ECG) with multiple rhythms. Electrophysiological study results showed sinus rhythm with APCs in bigeminal rhythm. When APCs were abolished by radiofrequency ablation, this patient was free from any arrhythmia during follow-up.

## 2 Case report

**Figure 1:**
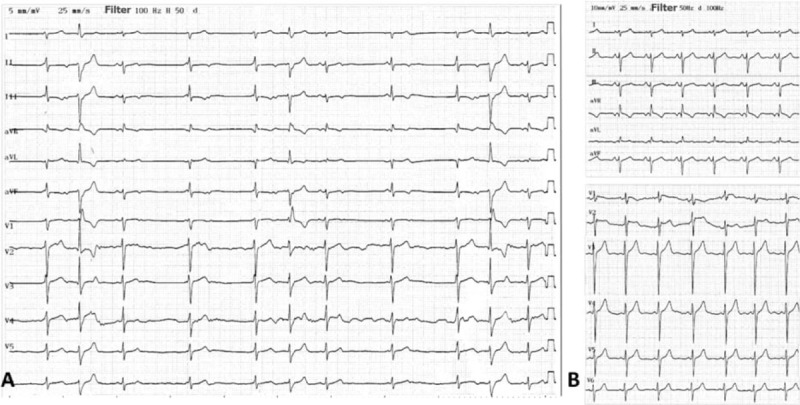
(A) Twelve-lead baseline ECG. (B) Twelve-lead ECG after ablation.

A 15-year-old male patient presented with recurrent paroxysmal palpitation for 6 months. Routine blood tests including thyroid function were normal. Echocardiography revealed no structural heart disease with normal left ventricular ejection fraction (66.1%). The baseline standard 12-lead ECG showed (Fig. 1A) multiple rhythms.

The written informed consent was obtained before the electrophysiological study. The electrophysiological study results showed sinus rhythm with nonconducted APCs in bigeminal rhythm. The AH interval was 60 ms and the HV interval was 67 ms during sinus rhythm and nonconducted APCs were blocked without H wave (Fig. 2A). Some APCs conducted to ventricle with AH interval 110 ms and the HV interval 75 ms (Fig. 2B). Detailed electroanatomic mapping of APCs was performed and guided by the 3-dimensional electroanatomic mapping system [CARTO 3 EP Navigation System, Biosense Webster (Israel) Ltd.]. The activation map showed that the earliest APC activation was at the ostium of coronary sinus. Radiofrequency ablation energy was delivered with 35 W, 43°C, and water flow velocity 17 mL/min at the ostium of coronary sinus (Fig. 2D). APCs were eliminated and ECG presented inerratic sinus rhythm (Fig. 2C). Further electrophysiological study found that the antegrade wenckebach block cycle length was 380 ms and the retrograde wenckebach cycle length was 420 ms during sinus rhythm. No tachycardia was induced by programmed stimulation. The resting ECG in the next day (Fig. 1B) and 2-week Holter monitor results showed sinus rhythm without APC or other arrhythmias.

**Figure:**
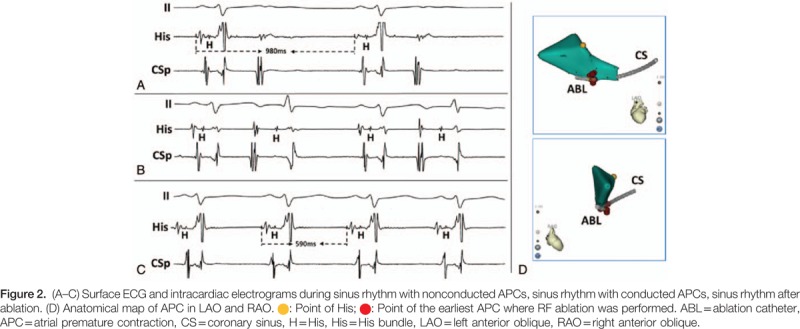


## 3 Discussion

**Figure 3:**
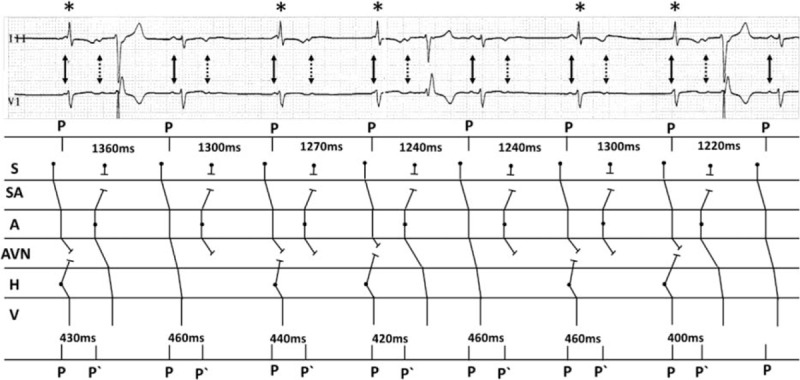
Ladder diagram to explain the mechanism of baseline ECG rhythm. Numbers on the top express the sinus p wave cycle lengths and numbers at the bottom are the PP‘ intervals on the ECG. P waves were marked with solid arrow, P‘ waves were marked with dashed arrow, and narrow QRS extrasystoles were marked with asterisks in the top strip. A = atrium, AVN = atrioventricular node, H = His bundle, P‘ = atrial extrasystole P wave, P = sinus P wave, S = sinus node, SA = sinoatrial junction, V = ventricle.

The baseline ECG of this case was very interesting and seemingly confused. First, in the baseline ECG, according to the P wave‘s morphology and electrical axis, the positive P waves (P) in Ш lead (marked with solid arrow) were deemed to sinus rhythm and the negative P waves (P‘) (marked with dashed arrow) were atrial extrasystoles, even though some of them were confused with QRS waves or T waves inordinately. The PP interval and PP‘ interval were seemed regularly (Fig. 3). The endocardial ECG presenting with APCs in bigeminal rhythm inerratic was in accord with the baseline ECG (Fig. 2A, B). The AA interval between 2 adjacent sinus beats with 1 APC in the middle before ablation was 980 ms (Fig. 2A). After ablation, the AA interval changed to 590 ms with APC disappearing (Fig. 2C). Bonke et al had elicited premature beats by electrical stimulation in right atrium of the rabbit and simultaneously put multiple microelectrode impalements of sinoatrial node fibers.^[1]^ They found that the impulse of the atrial premature beat excited the sinoatrial node only fractionally. Therefore, we thought that there was 2:1 S-A conduction because APCs appeared earlier than dormant sinus impulses and blocked it conducting to atrium (Fig. 3).^[2]^ In addition, we could not rule out the possibility that some APCs reached sinus node and reseted sinus rhythm partially. Second, baseline ECG showed regular narrow QRS complex extrasystoles (marked with asterisks in the top strip) that appeared after nonconducted APCs and confused with sinus P wave inordinately (Fig. 3). Endocardial ECG showed that nonconducted APCs failed to conduct to the ventricles without H wave and were blocked at the atrioventricular node level. The narrow QRS complex extrasystoles might originate from junctional atrioventricular junction region as compensatory rhythm of long interval without ventricular activation after nonconducted APCs. So, the retrograde conduction of junctional extrasystoles and the anterograde conduction of the sinus impulses were meanwhile blocked at AV node level. Then, baseline ECG presented narrow QRS wave confusing with sinus P wave (Fig. 3). When APCs conducted to ventricle, the narrow QRS complex extrasystole as a compensatory rhythm disappeared and adjacent sinus impulses could conduct to ventricle.^[3]^

Unfortunately, the narrow QRS extrasystoles did not appear during electrophysiological study, which might be because the patient was nervous during electrophysiological study without general anesthesia, which led to the rate of sinus rhythm faster than at resting time. Then, the atrioventricular junction extrasystoles were inhibited. Also, persistent atrial tachycardias can mimic atrial flutter, or even induce an atypical atrial flutter that requires careful differential diagnosis.

## 4 Conclusion

So, we considered that the basic rhythm of the baseline ECG was sinus rhythm with atrial bigeminy rhythm and narrow QRS extrasystoles (junctional); some APCs were blocked and some APCs conducted to ventricle with aberrant QRS complexes. When APCs were abolished by radiofrequency ablation, this patient was free from any arrhythmia during follow-up. Therefore, we considered that the phenomenon of baseline ECG was caused by the APCs.
